# Supplementary material for: The Role of Diet and Physical Activity in Shaping COVID-19 Severity: Design, Validation, and Application of a Retrospective Questionnaire
Source: Healthcare (Basel). 2024 Apr 10;12(8):813. doi: 10.3390/healthcare12080813 (PMC11050546; doi:10.3390/healthcare12080813)
Supplement: Supplementary file 1 [file healthcare-12-00813-s001.zip › healthcare-2921176-supplementary.pdf]

Supplementary Material. Lifestyle in Times of COVID-19 Questionnaire (Spanish)

ESTILO DE VIDA EN TIEMPOS DE COVID-19

El propósito de este estudio "Estilo de vida en tiempos de COVID-19" pretende conocer sus hábitos de actividad física y alimentación previo al diagnóstico de COVID-19 a través de este formulario, con la finalidad de generar recomendaciones generales para la prevención de la severidad por COVID-19. En este sentido, su contribución es muy valiosa por lo que le pedimos amablemente contestar con la mayor precisión.

Toda la información proporcionada en este formulario se manejará con estricta confidencialidad, es decir, sin revelar la identidad del participante, mismos que se utilizarán única y exclusivamente para fines académicos y de investigación, señalando que su participación es completamente voluntaria y no representa ningún riesgo físico, emocional o laboral.

Estoy de acuerdo en participar en el estudio y acepto los términos de privacidad. (https://www.gob.mx/indesol/documentos/ley-general-de-proteccion-de-datos-personales-en-posesion-de-sujetos-obligados)
-Acepto
-No acepto (termina el cuestionario)

INFORMACIÓN PERSONAL

Correo electrónico:
_____

Sexo:
-Hombre
-Mujer

Edad:
_____

Lugar de residencia:
_____

Origen étnico:
-Mestizo (mexicano)
-Europeo
-Africano
-Asiático
-Amerindio
-Caucásico (raza blanca)
-Mulato

Nivel de escolaridad:
-Posgrado
-Licenciatura

-Preparatoria
-Secundaria
-Primaria
-Sin estudio
-Otra:

Estado civil:
-Soltero
-Casado
-Otra:

Ocupación:
-Empleado
-Trabajo independiente
-Estudiante
-Desempleado
-Otra:

DIAGNÓSTICO DE COVID-19

En esta sección le solicitamos información acerca de cómo fue el diagnóstico y los síntomas que presentó por la enfermedad de COVID-19.

Propósito: Seleccionar a la población que puede participar en el estudio.

1.- Durante el periodo de pandemia, ¿Se ha enfermado de COVID-19?
-Si
-No (**termina el cuestionario**).
-No lo se

Propósito: Evaluar si el diagnóstico de COVID-19 se hizo a través de una prueba confirmatoria.

2.- Si usted enfermó de COVID-19, ¿Fue diagnosticado por un médico o realizó una prueba confirmatoria?
-Si
-No (pasa a la pregunta 4)

Propósito: Evaluar si el diagnóstico de COVID-19 se hizo a través de una prueba confirmatoria.

3.- Si realizó una prueba confirmatoria, ¿Cuál prueba diagnóstica le realizaron?
- PCR en tiempo real
- Prueba de detección de antígeno
- Prueba de detección de anticuerpos
- El médico me diagnóstico por los malestares o síntomas que presenté
-Otra:

Propósito: Conocer en qué periodo de la pandemia ocurrió la infección por Sars-Cov2 e inferir en la posible variante del virus.

4.- Si tuvo COVID-19 ¿En qué periodo de la pandemia enfermó la primera vez?:
-Desglose de mes y año

Propósito: Evaluar los síntomas más comunes que se presentaron durante la enfermedad de COVID-19.

5.- Marque los malestares o síntomas que presentó por infección de COVID-19:
-No presenté ningún malestar o síntoma (pasa a la pregunta 13)
-Dolor de cabeza
-Dolor de garganta
-Fiebre
-Dolor articular
-Dolor de ojos
-Cansancio
-Diarrea
-Vómito
-Dificultad para respirar o sensación de falta de aire
-Tos
-Pérdida del sentido del olfato y/o gusto
-Congestión nasal
-Escurrimiento nasal (gripe)
-Otra:

Propósito: Evaluar el tiempo de infección durante la enfermedad de COVID-19.

6.- La primera vez que tuvo COVID-19 ¿Cuántos días presentó los síntomas?:
_____

Propósito: Evaluar la severidad de la enfermedad de COVID-19.

7.- ¿Usted requirió ser hospitalizado?:
-Si
-No (pase a la pregunta 11)
-No necesité atención hospitalaria, solo acudí a un consultorio médico (pase a la pregunta 11)

Propósito: Evaluar el tipo de atención médica durante la infección por Sars-Cov2.

8.- ¿Qué tipo de atención hospitalaria/médica recibió?
-Privada
-Pública
-Otra:

Propósito: Evaluar la severidad de la enfermedad de COVID-19.

9.- Si fue hospitalizado, ¿Cuántos días permaneció en la unidad hospitalaria de salud?
_____

Propósito: Evaluar la severidad de la enfermedad de COVID-19.

10.- Si fue hospitalizado, ¿Fue necesario ingresar a la Unidad de Cuidados Intensivos (UCI)?
-Si
-No

Propósito: Evaluar la severidad de la enfermedad de COVID-19.

11.- ¿Requirió algún soporte de oxígeno en casa o en alguna unidad hospitalaria de salud?
-Si
-No (pasa a la pregunta 13)

Propósito: Evaluar la severidad de la enfermedad de COVID-19.

12.- Si requirió de oxígeno ¿Cuántos tiempo lo necesitó?
_____

Propósito: Evaluar la severidad de la enfermedad de COVID-19.

13.- ¿Cómo considera que fue la severidad de su enfermedad?
-Leve
-Moderada
-Severa
-Otra

Propósito: Conocer si durante la enfermedad de COVID-19 hubo pérdida o ganancia de peso

14.- Mencione su peso en kilogramos previo al diagnóstico de COVID-19 (Ej. 70):
_____

Propósito: Conocer si durante la enfermedad de COVID-19 hubo pérdida o ganancia de peso

15.- Mencione su peso en kilogramos después de enfermar de COVID-19 (Ej. 70):
_____

Propósito: Conocer si durante la enfermedad de COVID-19 hubo pérdida o ganancia de peso

16.- ¿Cuál es su estatura en metros? (Ej. 1.70)
_____

ENFERMEDADES PREEXISTENTES
En esta sección le solicitamos información acerca de sus antecedentes patológicos

Propósito: Evaluar si el sujeto presentaba alguna alteración metabólica que se pueda asociar a la severidad de COVID-19

17.- Previo a enfermar de COVID-19, mencione si presentaba alguna de las siguientes enfermedades:
-No tenía ninguna enfermedad (pasa a la pregunta 20)
-Obesidad

-Diabetes
-Hipertensión arterial
-Enfermedad renal crónica
-Diagnóstico de cáncer
-Enfermedad cardiaca
-Enfermedad obstructiva crónica (EPOC)
-Asma
-Enfermedad Autoinmune
-VIH/SIDA
-Enfermedades del hígado
-Otra

Propósito: Evaluar si el control metabólico de alguna enfermedad preexistente puede asociarse con la severidad de COVID-19

18.- En caso se presentar una enfermedad, ¿Cuenta con un control médico regular de la misma?
-Si
-No
-Acudo al médico solo cuando me siento mal

HÁBITOS DE ALIMENTACIÓN
¡ATENCIÓN! la información solicitada en esta sección hace referencia a los hábitos de alimentación previo a enfermar de COVID-19.

Propósito: Evaluar los hábitos de alimentación previo al diagnóstico de COVID-19 en la población encuestada.

20.- Seleccione los tiempos de comida que acostumbraba a realizar en el día antes de enfermar de COVID-19:
-Desayuno
-Comida
-Cena
-Colación 1
-Colación 2
-Otra:

Propósito: Evaluar los hábitos de alimentación previo al diagnóstico de COVID-19 en la población encuestada.

21.- Antes de enfermar, ¿Tenía horarios de alimentación establecidos?
-Si
-No
-Ocasional

Propósito: Evaluar los hábitos de alimentación previo al diagnóstico de COVID-19 en la población encuestada.

22.- Antes de enfermar, ¿Cómo considera que era su alimentación?
-Equilibrada
-Excesiva en azúcar
-Excesiva en grasa
-Baja en frutas y verduras
-Otra:

Propósito: Evaluar los hábitos de alimentación previo al diagnóstico de COVID-19 en la población encuestada.

23.- Antes de enfermar, ¿Con qué frecuencia consumías alimentos fuera de casa?
-Todos los días
-3 a 5 veces por semana
-1 a 2 veces por semana
-1 vez a la quincena
-1 vez al mes
-No acostumbraba a comprar
-Otra:

Propósito: Evaluar los hábitos de alimentación previo al diagnóstico de COVID-19 en la población encuestada.

24.- Antes de enfermar, ¿Qué cantidad de agua simple consumía al día?
-Más de 2 litros
- 1.5 – 2 litros
-1 – 1.5 litros
-Menos de 1 litro
-Otra:

Propósito: Evaluar hábitos de alimentación asociados con la severidad de la enfermedad.

25.- Antes de enfermar, ¿Con qué frecuencia consumía bebidas azucaradas industrializadas usando como referencia una lata de 355ml?
-No consumía
-1 a 2 veces por semana
-3 a 5 veces por semana
-Todos los días
-1 vez al mes
-Otra

Propósito: Evaluar hábitos de alimentación asociados con la severidad de la enfermedad.

26.- Antes de enfermar, ¿Cuántas porciones de productos industrializados consumía a la semana? (galletas, panadería, fritura y/o caramelos, etc.)
-Ninguno
-Todos los días al menos una porción
-3 veces por semana al menos una porción
-Una vez por semana al menos una porción

-Otro:

Propósito: Evaluar hábitos de alimentación asociados con la severidad de la enfermedad.

27.- Antes de enfermar, ¿Cuántas cucharadas de azúcar añadida agregabas a tus alimentos y/o bebidas?

_____

Propósito: Evaluar los hábitos de alimentación previo al diagnóstico de COVID-19 en la población encuestada.

28.- Antes de enfermar de COVID-19, ¿Cuántas piezas de frutas consumías al día?
-No consumo
-1-2 porciones
-3-5 porciones
-Otra:

Propósito: Evaluar los hábitos de alimentación previo al diagnóstico de COVID-19 en la población encuestada.

29.- Antes de enfermar de COVID-19, ¿Cuántas piezas de verduras consumías al día?
-No consumo
-1-2 porciones
-3-5 porciones
Otra:

Propósito: Evaluar los hábitos de alimentación previo al diagnóstico de COVID-19 en la población encuestada.

30.- Antes de enfermar, ¿Cuántas veces por semana consumía carne roja y/o embutidos?
-1-2 veces
-3-5 veces
-Todos los días
-1 vez por quincena
-1 vez al mes
-No consumo

Propósito: Evaluar los hábitos de alimentación previo al diagnóstico de COVID-19 en la población encuestada.

31.- Antes de enfermar, ¿Cuántas veces por semana consumía pescado blanco y/o mariscos?
-No consumo
-1-2 veces
-3-5 veces
-Todos los días
-1 vez por quincena
-1 vez al mes

Propósito: Identificar aspectos del estilo de vida previo al diagnóstico de COVID-19 que se relacionen con la severidad de la enfermedad.

32.- Fumaba tabaco:
-Si
-No (pasar a pregunta 34)
-Ocasional

Propósito: Identificar aspectos del estilo de vida previo al diagnóstico de COVID-19 que se relacionen con la severidad de la enfermedad.

33.- Antes de enfermar, ¿Cuántos cigarrillos fumabas al día?
_____

Propósito: Identificar aspectos del estilo de vida previo al diagnóstico de COVID-19 que se relacionen con la severidad de la enfermedad.

34.- Antes de enfermar, ¿Consumía alcohol?
-Si
-No (pasar a pregunta 37)
-Ocasional

Propósito: Identificar aspectos del estilo de vida previo al diagnóstico de COVID-19 que se relacionen con la severidad de la enfermedad.

35.-Antes de enfermar, ¿Con que frecuencias consumía alcohol?
-Diario
-Semanal
-Mensual
-Ocasional

Propósito: Identificar aspectos del estilo de vida previo al diagnóstico de COVID-19 que se relacionen con la severidad de la enfermedad.

36.- Cuando consumía alcohol, ¿Qué cantidad de alcohol consumía tomando en cuenta 1 lata de 355ml?
-2-5 latas
-más de 5 latas
-Otra:

Propósito: Identificar aspectos del estilo de vida previo al diagnóstico de COVID-19 que se relacionen con la severidad de la enfermedad.

37.- Antes de enfermar, ¿Cuántas horas acostumbraba a dormir la noche?
-6 a 8 horas
-Menos de 6 horas
-Más de 8 horas
-Otra:

Propósito: Identificar aspectos del estilo de vida previo al diagnóstico de COVID-19 que se relacionen con la severidad de la enfermedad.

38.- Antes de enfermar. Al despertar, ¿Se levantaba con sensación de descanso?
-Si
-No
-A veces

ACTIVIDAD FÍSICA
Estamos interesados en saber acerca del nivel de actividad física relacionada al deporte que usted hace de manera regular.

¡ATENCIÓN! la información solicitada en esta sección hace referencia a la actividad física que realizabas previo a enfermar de COVID-19.

Propósito: Evaluar el nivel de actividad física relacionada al deporte que realizaban de manera regular previo al diagnóstico de COVID-19 y su posible asociación con la recuperación de la enfermedad.

39.- Antes de enfermar, ¿Qué tipo de actividad física realizaba?
-No realizaba ningún tipo de ejercicio (pasar a la pregunta 44)
-Gimnasio
-Caminata
-Correr
-Ciclismo
-Zumba/Aeróbico
-Spining
-Tenis
-Natación
-Fútbol
-Baile
-Atletismo
-Otra:
Propósito: Evaluar el nivel de actividad física relacionada al deporte que realizaban de manera regular previo al diagnóstico de COVID-19 y su posible asociación con la recuperación de la enfermedad.

40.- ¿Cuántos días de la semana realizaba actividad física?
-Diario
-5 a 6 días
-3 a 4 días
-Menos de dos días
Otra:

Propósito: Evaluar el nivel de actividad física relacionada al deporte que realizaban de manera regular previo al diagnóstico de COVID-19 y su posible asociación con la recuperación de la enfermedad.

41.- ¿Cuánto tiempo realizaba actividad física?
- Más de 50 minutos por sesión
- 30 a 45 minutos por sesión
- Menos de 30 minutos por sesión
-Otra:

Propósito: Evaluar el nivel de actividad física relacionada al deporte que realizaban de manera regular previo al diagnóstico de COVID-19 y su posible asociación con la recuperación de la enfermedad.

42.- ¿A qué intensidad realizaba la actividad física?
- Ligero (puede hablar y cantar)
- Ligero-moderado (puede hablar, pero no cantar)
- Moderado (su corazón late un poco más rápido de lo normal)
- Moderado-intenso (su corazón late más rápido de lo normal)
- Intenso (su corazón late fuerte, aparecen respiraciones profundas)
- Muy intenso (El número de latidos de su corazón aumenta mucho más rápido de lo normal, aparece la sensación de falta de aire)

Propósito: Evaluar el nivel de actividad física relacionada al deporte que realizaban de manera regular previo al diagnóstico de COVID-19 y su posible asociación con la recuperación de la enfermedad.

43.- ¿Cuánto tiempo llevabas entrenando? (Ejemplo. 2 años):

ACTIVIDAD FÍSICA
Estamos interesados en saber acerca del nivel de actividad física que usted realiza como parte de su vida diaria. Por favor responda cada pregunta aún si usted no se considera una persona activa. Piense en aquellas actividades que realiza como parte del trabajo, en el jardín, en la casa, en el transporte y en su tiempo libre de descanso.

¡ATENCIÓN! la información solicitada en esta sección hace referencia a la actividad física que realizabas previo a enfermar de COVID-19.

Propósito: Evaluar el nivel de actividad física relacionada a la vida diaria previo al diagnóstico de COVID-19

44.- Piense acerca de todas aquellas actividades intensas que usted realizó al menos una semana antes de enfermar de COVID-19. Actividades intensas son las que requieren un esfuerzo físico fuerte y le hacen respirar mucho más fuerte que lo normal. ¿Cuántos días realizó actividades físicas intensas como levantar objetos pesados, excavar o equivalente a excavar, aeróbicos, o pedalear rápido en bicicleta, etc.?
-1 día
-2 días
-3 días
-4 días
-5 días
-6 días

-Toda la semana
-Ningún día

Propósito: Evaluar el nivel de actividad física relacionada a la vida diaria previo al diagnóstico de COVID-19

45.- ¿Cuánto tiempo en total usualmente le tomó realizar actividades físicas intensas en uno de esos días que las realizó?:

_____

Propósito: Evaluar el nivel de actividad física relacionada a la vida diaria previo al diagnóstico de COVID-19

46.- Piense acerca de todas aquellas actividades moderadas que usted realizó al menos una semana antes de enfermar de COVID-19. Actividades moderadas son aquellas que requieren un esfuerzo físico moderado y hace que su corazón late un poco más rápido de lo normal. ¿Cuántos días realizó actividades físicas moderadas tal como cargar objetos livianos, pedalear en bicicleta a paso regular, o nadar, etc.? No incluya caminatas.
-1 día
-2 días
-3 días
-4 días
-5 días
-6 días
-Toda la semana
-Ningún día

Propósito: Evaluar el nivel de actividad física relacionada a la vida diaria previo al diagnóstico de COVID-19

47.- ¿Cuánto tiempo dedicó usted en uno de esos días realizar actividades físicas moderadas?

_____

Propósito: Evaluar el nivel de actividad física relacionada a la vida diaria previo al diagnóstico de COVID-19

48.- Piense acerca del tiempo que usted dedicó a caminar al menos una semana antes de enfermar de COVID-19. Esto incluye trabajo en la casa, caminatas para ir de un sitio a otro, o cualquier otra caminata que usted hizo únicamente por recreación, deporte, ejercicio, o placer. ¿Cuántos días caminó usted por al menos 10 minutos continuos?
-1 día
-2 días
-3 días
-4 días
-5 días
-6 días
-Toda la semana
-Ningún día

Propósito: Evaluar el nivel de actividad física relacionada a la vida diaria previo al diagnóstico de COVID-19

49.- ¿Cuánto tiempo gastó usted en uno de esos días caminando?:

_____

Propósito: Evaluar el nivel sedentarismo previo al diagnóstico de COVID-19 y su asociación con la severidad de la enfermedad.

50.- Piense en el tiempo que usted permaneció sentado(a) al menos una semana antes de enfermar de COVID-19. Incluya el tiempo sentado(a) en el trabajo, la casa, estudiando, y en su tiempo libre. Puede ser en un escritorio, visitando amigos(as), leyendo o permanecer sentado(a) o acostado(a) mirando televisión. ¿Cuánto tiempo permaneció sentado(a) en un día en la semana?

_____

Propósito: Evaluar el nivel de actividad física relacionada a la vida diaria previo al diagnóstico de COVID-19

51.- Las preguntas anteriores hacen referencia a una semana antes de enfermar de COVID-19. Sin embargo, si usted considera que este patrón de actividad física ha persistido por más tiempo en su vida diaria, nos interesa saber desde hace cuánto tiempo:

_____

VACUNACIÓN

Casi llegamos al final. ¡Gracias por continuar contestando!

Propósito: Conocer el tipo de vacuna y las dosis recibidas durante el periodo de pandemia y su posible asociación con los síntomas y severidad de la enfermedad.

52.- ¿Recibiste alguna vacuna contra-Covid19?

-Si
-No (terminar el cuestionario con el mensaje de agradecimiento)

Propósito: Conocer el tipo de vacuna y las dosis recibidas durante el periodo de pandemia y su posible asociación con los síntomas y severidad de la enfermedad.

53.- ¿Qué vacuna recibiste en tu primera dosis?

-Pfizer-BioNTech
-Moderna
-Johnson & Johnson
-CanSino
-AstraZeneca
-Sputnik V
-Sinovac
-Otra:

Propósito: Conocer el tipo de vacuna y las dosis recibidas durante el periodo de pandemia y su posible asociación con los síntomas y severidad de la enfermedad.

54.- ¿Qué vacuna recibiste en la segunda dosis?
-Pfizer-BioNTech
-Moderna
-Johnson & Johnson
-CanSino
-AstraZeneca
-Sputnik V
-Sinovac
-Otra:

Propósito: Conocer el tipo de vacuna y las dosis recibidas durante el periodo de pandemia y su posible asociación con los síntomas y severidad de la enfermedad.

55.- ¿Qué vacuna recibiste en la tercera dosis?
-Pfizer-BioNTech
-Moderna
-Johnson & Johnson
-CanSino
-AstraZeneca
-Sputnik V
-Sinovac
-Otra:

Propósito: Conocer el tipo de vacuna y las dosis recibidas durante el periodo de pandemia y su posible asociación con los síntomas y severidad de la enfermedad.

56.- ¿Usted se enfermó de COVID-19 antes o después de recibir su primera vacuna?
-Antes de la vacuna
-Después de la vacuna
-No estoy seguro

¡GRACIAS POR LLEGAR HASTA AQUÍ!
Para nosotros su participación es muy valiosa y nos gustaría retribuir su tiempo enviándole recomendaciones nutricionales generales para fortalecer su sistema inmune en tiempos de COVID-19. De *click* en el siguiente enlace para descargarlas: ***LINK***
Cualquier duda o comentario puede comunicarse al correo estilodevidaycovid@gmail.com
